# A New Ex Vivo Model Based on Mouse Retinal Explants for the Study of Ocular Toxoplasmosis

**DOI:** 10.3390/pathogens13080701

**Published:** 2024-08-19

**Authors:** Veronica Rodriguez Fernandez, Rosario Amato, Simona Piaggi, Barbara Pinto, Giovanni Casini, Fabrizio Bruschi

**Affiliations:** 1 Department of Translational Research, School of Medicine, University of Pisa, 56126 Pisa, Italy; 2 Department of Infectious Diseases and Public Health, La Sapienza University, 00185 Rome, Italy; 3 Department of Biology, University of Pisa, 56126 Pisa, Italy; 4 Interdepartmental Research Center Nutrafood “Nutraceuticals and Food for Health”, University of Pisa, 56126 Pisa, Italy

**Keywords:** *T. gondii*, parasite infection, inflammation, oxidative stress, cell death

## Abstract

Ocular toxoplasmosis is the most prevalent clinical manifestation of *T. gondii* infection, which causes irreversible retinal damage. Different experimental models have been developed to study this pathology. In the present study, a new, ex vivo model is proposed to contribute to the elucidation of disease mechanisms and to possible therapeutic solutions. Ex-vivo retinal explants, prepared from mouse retinas following established protocols, were incubated with *T. gondii* tachyzoites maintained in Vero cells. At different times, starting at 12 h up to 10 days of incubation, the explants were analyzed with immunofluorescence and Western blot to investigate their responses to parasite infection. *T. gondii* invasion of the retinal thickness was evident after 3 days in culture, where parasites could be detected around retinal cell nuclei. This was paralleled by putative cyst formation and microglial activation. At the same time, an evident increase in inflammatory and oxidative stress markers was detected in infected explants compared to controls. Cell death also appeared to occur in retinal explants after 3 days of *T. gondii* infection, and it was characterized by increased necroptotic but not apoptotic markers. The proposed model recapitulates the main characteristics of *T. gondii* retinal infection within 3 days of incubation and, therefore, allows for studying the very early events of the process. In addition, it requires only a limited number of animals and offers easy manipulation and accessibility for setting up different experimental conditions and assessing the effects of putative drugs for therapy.

## 1. Introduction

*Toxoplasma gondii* is a widespread obligate intracellular parasitic protozoan responsible for toxoplasmosis. The host–parasite relationship is particularly intriguing at the ocular level; in fact, *T. gondii* manipulates the immune response and induces variable initial lesions and further relapses (ocular toxoplasmosis, OT), which ultimately may lead to blindness [1]. To prevent this, it is important to use control measures, but when the infection occurs, the diagnosis should be performed promptly to provide for treatment and/or monitoring [2].

OT is a vision-threatening disease and the most prevalent clinical manifestation of *T. gondii* infection, with different rates depending on the geographical region. For example, in Brazil, around 17% of the population experiences it, whereas in the United States, it affects approximately 2% of individuals [3]. OT usually results in the infection of the retina, causing irreversible retinal damage [4]. In particular, we may distinguish an active form of OT, characterized by the rapid replication of *T. gondii*’s tachyzoites within the retina, where they induce reactive inflammation, and a chronic form, which is the result of host cell destruction that leads to necrotic retinitis in the affected eye, often accompanied by vitritis and choroiditis [5].

Limited information is available regarding the response to infection of specific cellular types within the retina, and whether various retinal cell types exhibit different susceptibilities to infection remains unclear. There is also a gap in knowledge concerning the molecular mechanisms responsible for cell damage or death. Therefore, there is a need for new models that are easily manageable, allowing the modification of various infection parameters and the environment of the retina. Different in vitro and in vivo experimental models of ocular infection with *T. gondii* have been developed in past years [4,6], while an underexplored method is the utilization of organotypic, ex vivo retinal explants. Indeed, very few studies, to our knowledge, have proposed ex vivo models, constituted by either retinal explants from chick embryos to study parasite proliferation patterns [7] or post-mortem human eyecups infected with *T. gondii* tachyzoites to investigate parasite dissemination through the retinal layers [8]. Although these studies have provided valuable data with respect to the chick embryo, a model based on a mammalian retina appears preferable, while studies on human specimens are affected by ethical and practical issues. An ex vivo model of the mouse retina has been used previously in basic investigations of retinal disease [9,10,11,12,13,14]. In the present study, we characterized this model for the study of OT.

## 2. Materials and Methods

### 2.1. Host Cells Culture Conditions and T. gondii Maintenance

VERO cells (African green monkey kidney tissue cells, ATCC, CCL-81) were used as host cells for in vitro *T. gondii* maintenance and as control cells. Cells were cultured in Dulbecco’s modified minimum essential medium (DMEM)-α supplemented with 5% inactivated fetal bovine serum and 2 mM L-glutamine (Gibco, Rockville, MD, USA). The RH strain of *T. gondii* used in these studies was kindly provided by Furio Spano of the Italian National Institute of Health, Rome, Italy, and was maintained following previously established protocols [15].

Briefly, tachyzoites were maintained in vitro through serial passages in 25 cm^2^ cell culture flasks of confluent VERO cells. For the infection, parasites were stained with Trypan blue to assess viable and not viable tachyzoites and quantified using a Bürker chamber under a light microscope (Carl Zeiss, Jena, Germany) (40× objective lens) and used to infect the cells (parasite/cell ratio = 5:1). The VERO cells were routinely sub-cultured every 3 days by trypsinization of confluent monolayers.

To isolate *T. gondii* tachyzoites from infected VERO cells, we collected them once the VERO cells were lysed by the parasites. The medium was centrifuged at 800× *g* for 4 min, and the supernatant was collected to remove cellular debris. The clarified supernatant was then subjected to a second centrifugation at 3500× *g* for 10 min. The resulting pellet containing *T. gondii* was resuspended in the same culture medium used for the VERO cells. Tachyzoites were then counted, and a new flask of VERO cells was subsequently infected.

### 2.2. Retinal Explants

Ex vivo retinal explants were prepared following previously established protocols [9] using C57BL/6J mice aged 3–6 weeks. All procedures were approved by the Commission for Animal Wellbeing of the University of Pisa (permission number 0034612/2017). They adhered to the ARVO Statement for the Use of Animals in Ophthalmic and Vision Research, the Italian animal care guidelines (DL 26/14), and the EU Directive (2010/63/EU). The mice were sacrificed by cervical dislocation, and the ocular globes were extracted. The retinas were dissected in MEM, divided into 4 fragments, and placed on Millicell-CM culture inserts (Merck Millipore, Darmstadt, Germany) with ganglion cells facing upward. The inserts, accommodating six fragments each, were positioned in 6-well tissue culture plates containing 1 mL of culture medium, as described in [9]. The explants were maintained at 37 °C in an atmosphere of 5% CO_2_. Each experimental condition was run in triplicate.

### 2.3. Retinal Explant Infection with T. gondii

**Figure 1:**
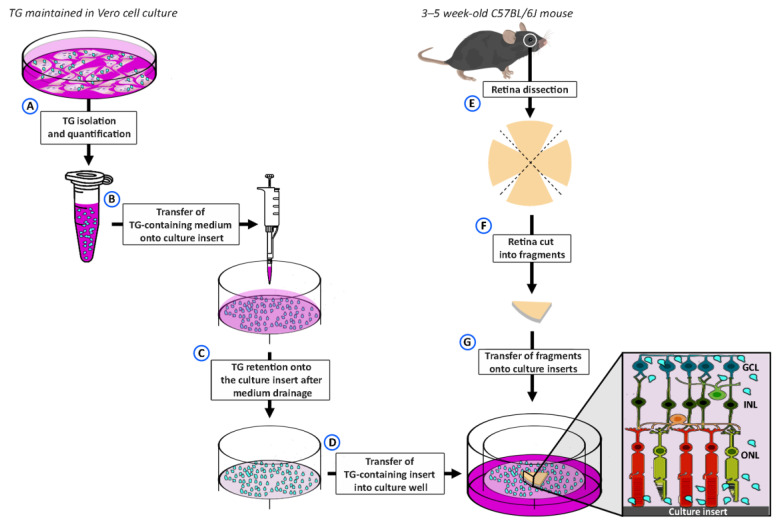
Procedure for *T. gondii* infection of cultured retinal explants. *T. gondii* (TG) from Vero cell cultures were isolated and counted (**A**). Aliquots of 2,000 or 20,000 TG were suspended in 500 μL of culture medium (**B**) and transferred onto the membrane of a culture insert. Due to the small pore size of the insert membrane (0.4 µm diameter), the medium was drained while TG remained on the membrane (**C**). The TG-containing insert was then moved into a culture well (**D**) containing 1 mL of culture medium. Retinal explants were prepared from retinas of 3–5-week-old C57BL/6J mice (**E**). Each retina was divided into four fragments (**F**), and the fragments (retinal explants) were positioned ganglion cells up onto the membrane of the culture insert (**G**). A total of six explants were laid in each insert (although only one is shown in the figure). See the text for a more detailed description.

Retinal explants were infected with 2000 *T. gondii*/well (approximate number evaluated by optical microscope counting, using the Bürker chamber). This dose was chosen according to our experience in in vitro infections of mammalian cells and to preliminary experiments with ex vivo retinal explants in which we used larger amounts of parasites. Since the obtained results were similar to different doses (from 2000 to 20,000 *T. gondii*/well), we chose to use the smaller amount to mimic the in vivo exposure of the retina to invading parasites as much as possible. The parasites were suspended in 500 μL of the culture medium used for retinal explants. Then, 500 μL of culture medium alone was used for control experiments. As summarized in Figure 1, the 500 μL was poured onto Millicell CM culture inserts, whose membranes are characterized by pores of 0.4 μm in size (product data sheet). Since *T. gondii* dimensions are larger than those of these pores, the parasite was retained on the insert membrane, while the culture medium was drained through the membrane by gravity. The insert was then transferred into a culture well containing 1 mL of culture medium, a volume that did not cover the insert but was sufficient to touch the membrane from below and form a thin layer on its surface. Retinal explants obtained as described above were positioned onto the culture insert (ganglion cells up) and the medium containing the parasite and wrapped around the explant by capillarity. In this way, both sides of the explant were exposed to and could come into contact with *T. gondii*. The retinal explants were incubated for 12 h, 1 day, 3 days, 7 days, or 10 days. The culture medium was changed every other day. After incubation, the retinal explants were used for either immunofluorescence or Western Blotting.

### 2.4. Immunofluorescence

Vero cells were fixed in 4% paraformaldehyde in 0.1 M phosphate-buffered saline (PBS) for 1 h at room temperature, washed in PBS, and incubated with an antibody directed to *T. gondii* (see Table 1) at a dilution of 1:500 followed by incubation with Alexa Fluor 488-conjugated secondary antibody (Life Technologies, Carlsbad, CA, USA, 1:200 dilution) for 1 h. The retinal explants were fixed in 4% paraformaldehyde in 0.1 M PB for 2 h at room temperature, followed by storage in 25% sucrose in PB at 4 °C. For cryostat sectioning, the fixed explants were embedded in cryo-gel (Killik, O.C.T. Compound, Bio-Optica, Milano, Italy), rapidly frozen using liquid nitrogen, and cut into 10 μm-thick coronal sections. The sections were then mounted onto gelatin-coated slides and stored at −20 °C. For immunostaining, sections were incubated overnight at 4 °C with the antibodies listed in Table 1, followed by incubation in appropriate Alexa Fluor 488- and/or Alexa Fluor 546-conjugated secondary antibodies (1:200 dilution) at room temperature for 2 h. The slides with either VERO cells or retinal sections were washed and coverslipped using Fluoroshield Mounting Medium containing DAPI (Abcam, Cambridge, UK). Immunofluorescence images were captured with an epifluorescence microscope (Nikon Europe, Amsterdam, The Netherlands) and adjusted for contrast and brightness using Adobe Photoshop (Adobe Photoshop CS3; Adobe Systems, Mountain View, CA, USA).

### 2.5. Western Blotting

Total protein extraction was performed using RIPA Lysis buffer supplemented with protease and phosphatase inhibitor cocktails. Protein concentration was quantified using a fluorometer (Qubit; Invitrogen, Carlsbad, CA, USA). Equal amounts of protein (30 μg) were separated using SDS-PAGE and transferred onto polyvinylidene difluoride membranes (Bio-Rad Laboratories, Inc., Hercules, CA, USA). The membranes were blocked in 3% skim milk for 1 h and then incubated overnight with the antibodies listed in Table 2 followed by incubation with horseradish peroxidase-conjugated anti-rabbit (Bio-Rad, 1:5000 dilution) or anti-mouse (Merck Sigma-Aldrick, 1:5000 dilution) secondary antibodies for 1 h at room temperature. The blots were developed by the Clarity Western-enhanced chemiluminescence substrate, and the signal was detected using ChemiDoc XRS+ (Bio-Rad). The band optical densities were assessed using Image Lab 3.0 software (Bio-Rad). The data were normalized to the corresponding OD of β-actin or to that of NF-kB p65. All experiments were conducted in triplicate.

### 2.6. Statistics

The data of Western blotting were analyzed using one-way analysis of variance (ANOVA) followed by Tukey’s post hoc test. The results were expressed as mean ± SEM of the indicated *n* values (Prism 8; GraphPad software, San Diego, CA, USA). Differences with *p* < 0.05 were considered significant.

## 3. Results

Infected Vero cells were immunostained with antibody Thermofisher PA1-7256 to test the reactivity against *T. gondii*. As shown in supplementary Figure S1, the parasites were clearly identified in infected cultures, while control cultures were negative.

**Figure 2:**
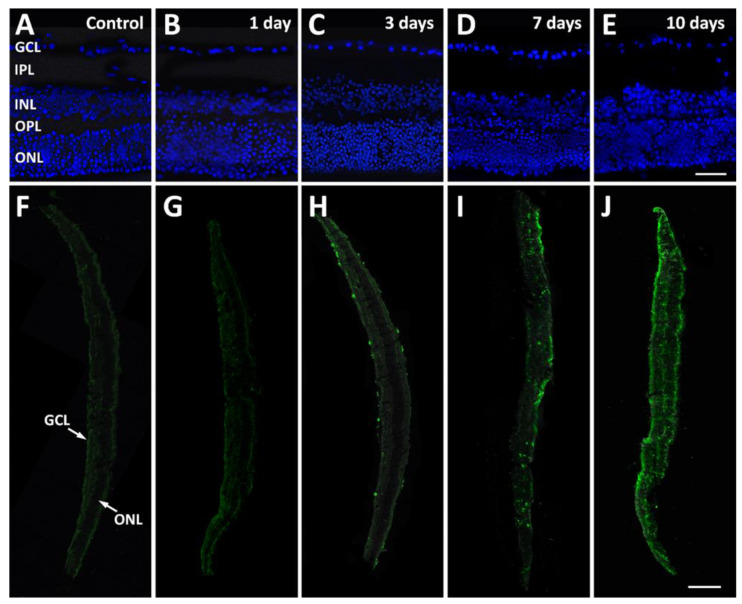
Representative DAPI-stained sections from control retinal explants (**A**) and from explants after 1 day (**B**), 3 days (**C**), 7 days (**D**), and 10 days (**E**) of incubation in the presence of *T. gondii* showing only minor alterations of retinal layer organization throughout the culture period. Scale bar, 50 μm. (**F**–**J**): Representative low-power photomicrographs of sections immunolabeled with an antibody directed to *T. gondii* and obtained from control retinal explants (**F**) and from explants after 1 day (**G**), 3 days (**H**), 7 days (**I**), and 10 days (**J**) of incubation in the presence of *T. gondii*. Scale bar: 20 μm. Abbreviations: GCL, ganglion cell layer; INL, inner nuclear layer; IPL, inner plexiform layer; ONL, outer nuclear layer; OPL, outer plexiform layer.

Retinal explants remained viable in culture for up to 10 days with no appreciable changes in the architecture or in the relative thickness of the retinal layers, from the ganglion cell layer (GCL), containing the ganglion cell somata, to the outer nuclear layer (ONL), or containing the cell bodies of photoreceptors (Figure 2A–E).

### 3.1. Infection of Retinal Explants by T. gondii: Time Course and Microglia Activation

**Figure 3:**
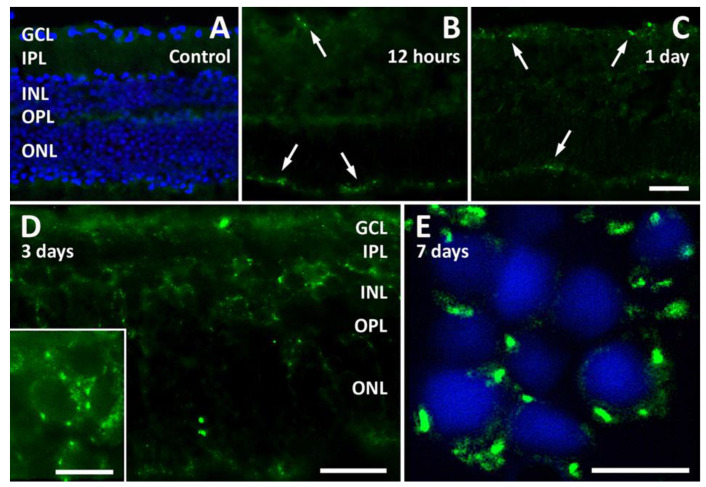
Representative photomicrographs of sections from retinal explants immunolabeled with an antibody directed to *T. gondii*. (**A**–**C**): Immunolabeled sections from control retinal explants ((**A**), with DAPI counterstain) and from explants after 12 h (**B**) and 1 day (**C**) of incubation in the presence of *T. gondii*. The arrows point to *T. gondii* immunofluorescence localized superficially to both the GCL and the ONL sides of the explant. Scale bar, 50 μm. (**D**,**E**): Higher-power photomicrographs of retinal sections from retinal explants after 3 days and 7 days, respectively, of incubation with *T. gondii* ((**E**) with DAPI counterstain). Scale bars: (**D**) 50 μm (inset, 10 μm); (**E**) 10 μm. See Figure 2 for abbreviations.

In low-power immunofluorescence images, the parasite appeared at both the inner (GCL side) and outer (ONL side) edges of retinal explants after 3 days in culture, while the invasion of internal retinal layers was evident after 7 or 10 days (Figure 2F–J). At higher power, while control explants were devoid of immunolabeling (Figure 3A), a few parasites were seen at both retinal edges as early as after 12 h in culture (Figure 3B), and a similar picture was maintained after 1 day (Figure 3C). At 3 days, parasites had reached the inside of the retinal tissue (Figure 3D) and had infected retinal cells in the inner nuclear layer (INL) and in the ONL (Figure 3D, inset). Retinal cell infection was clearly visible in DAPI-counterstained retinal sections from explants after 7 days in culture, where a bright immunofluorescent signal indicated the presence of *T. gondii* all around cell nuclei (Figure 3E).

**Figure 4:**
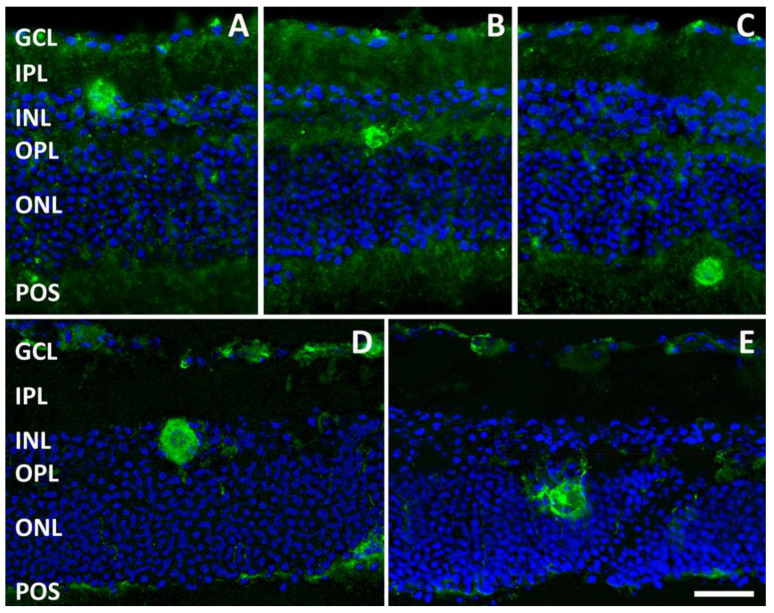
Representative photomicrographs of sections from retinal explants immunolabeled with an antibody directed to *T. gondii* (and counterstained with DAPI) showing the presence of immunolabeled putative cysts localized to different retinal layers after 3 days (**A**–**C**) and 7 days (**D**,**E**) of incubation with *T. gondii*. Scale bar: 50 μm. POS, photoreceptor outer layer. See Figure 2 for other abbreviations.

Immunofluorescent profiles indicating the possible presence of cysts formed by *T. gondii* bradyzoites were observed in retinal explants after 3 days (Figure 4A–C), 7 days (Figure 4D,E), and 10 days in culture. These putative cysts did not appear to have preferred retinal localizations, but they were observed in different retinal layers, including the inner plexiform layer (IPL), INL, outer plexiform layer (OPL), ONL, and the layer of the photoreceptor outer segments (POS).

**Figure 5:**
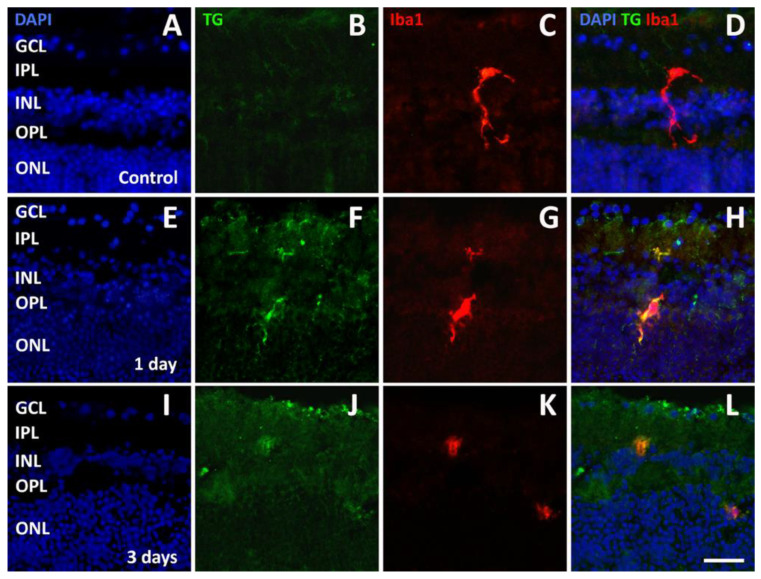
Representative photomicrographs of sections double-labeled with antibodies directed to *T. gondii* (TG, green) and ionized calcium-binding adapter molecule 1 (Iba1, red) from control retinal explants (**A**–**D**) and from explants after 1 day (**E**–**H**) and 3 days (**I**–**L**) of incubation in the presence of *T. gondii*. The sections were counterstained with DAPI. Scale bar, 50 μm. See Figure 2 for abbreviations.

Microglial cells identified with ionized calcium-binding adapter molecule 1 (Iba1) immunofluorescence were observed in control retinal explants (Figure 5A–D). They were mainly localized within the IPL and the OPL and were characterized by long and ramified processes. After 1 day in culture with *T. gondii*, Iba-1 immunofluorescent microglial cells could be observed not only in the plexiform layers but also in the INL or in the ONL. At this stage, these cells displayed shorter and less ramified processes, typical of reactive or activated microglial cells [16]. Notably, they were double-labeled by antibodies directed to *T. gondii*, likely indicating phagocytosis of the parasite by microglial cells (Figure 5E–H). After 3 days in culture, Iba1 immunolabeled microglial cells appeared to have completely lost their ramified processes, assuming an amoeboid morphology. They had no specific localization in retinal layers and were still double-labeled by *T. gondii* antibodies (Figure 5I–L). These characteristics were maintained in explants examined after 7 or 10 days in culture.

These data depicting the time course of *T. gondii* invasion of retinal layers and of microglia activation indicated that acute infection is achieved within 3 days of incubation. Therefore, the following analyses concentrated on this time window to provide a comprehensive characterization of the model.

### 3.2. Effects of T. gondii Infection on Inflammation and Oxidative Stress

**Figure 6:**
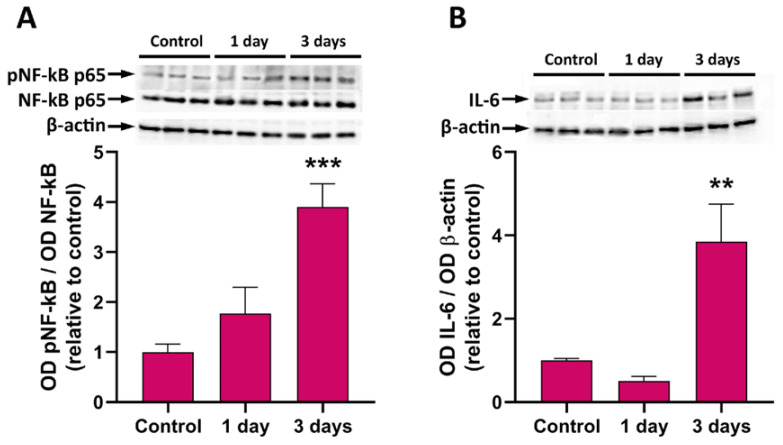
Western blot analysis showing representative immunoreactive bands and quantitative densitometric analysis of pNF-kB p65/NF-kB p65 ratio (**A**) and of IL-6 protein levels (**B**) in control retinal explants and from explants after 1 day and 3 days of incubation in the presence of *T. gondii*. ** *p* < 0.01; *** *p* < 0.001.

The level of inflammation was evaluated using Western blotting for two commonly used inflammatory markers, NF-kB phosphorylation and IL-6 levels. Both NF-kB phosphorylation and the content of IL-6 increased in retinal explants, reaching significantly higher levels than in controls after 3 days in culture (Figure 6).

**Figure 7:**
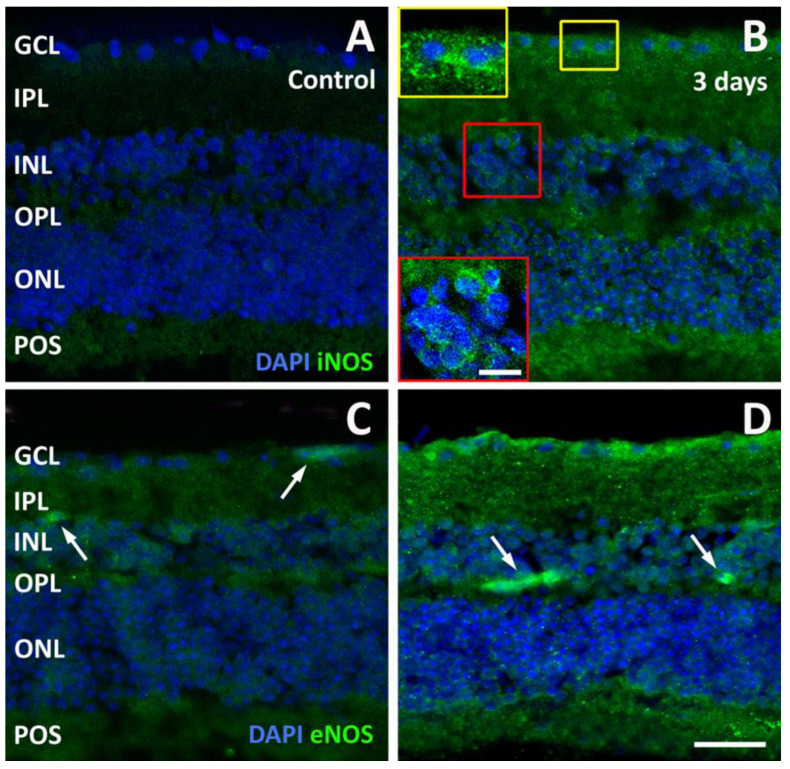
Representative photomicrographs of sections immunolabeled with an antibody directed to inducible nitric oxide synthase (iNOS, (**A**,**B**)) or endothelial NOS (eNOS, (**C**,**D**)) from control retinal explants (**A**,**C**) and from explants after 3 days of incubation in the presence of *T. gondii* (**B**,**D**). The sections were counterstained with DAPI. The arrows in (**C**,**D**) point to eNOS-immunolabeled blood capillaries. Scale bar, 50 μm (inset, 20 μm). See Figure 4 for abbreviations.

Together with an increase in NF-kB phosphorylation and IL-6 production, a hallmark of retinal inflammation is the rise in inducible nitric oxide synthase (iNOS). As shown in Figure 7A, control explants were almost devoid of iNOS immunofluorescence, while iNOS immunolabeling was clearly detectable in explants after 3 days of incubation with *T. gondii* (Figure 7B), where it was particularly densely distributed within the cytoplasm of cells in the GCL and in the INL (Figure 7B, insets). We also examined the localization patterns of another form of NOS, endothelial NOS (eNOS). In control explants, eNOS immunofluorescence was mainly limited to blood vessels (Figure 7C), but in explants after 3 days of incubation, eNOS immunofluorescence in blood vessels appeared much more intense, and it was also observed in the GCL (Figure 7D).

**Figure 8:**
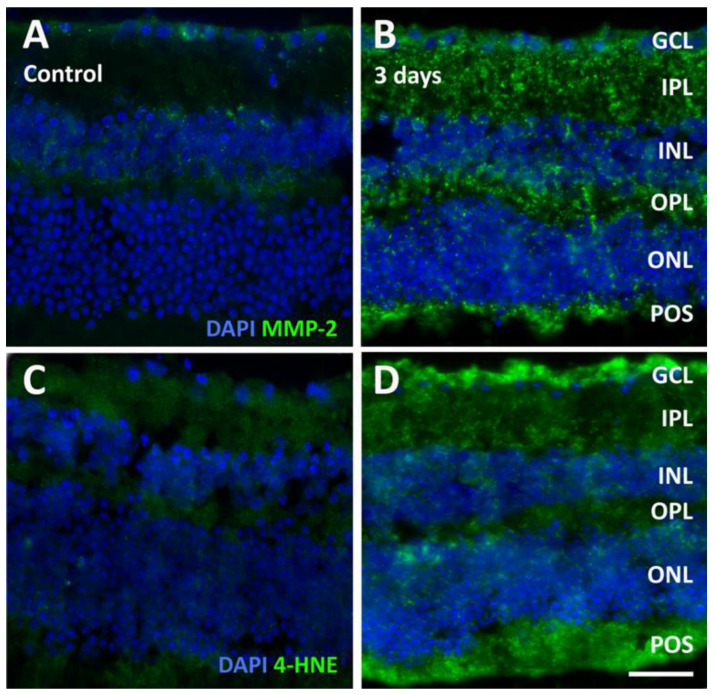
Representative photomicrographs of sections immunolabeled with an antibody directed to matrix metalloproteinase-2 (MMP-2, (**A**,**B**)) or 4-hydroxynonenal (4-HNE, (**C**,**D**)) from control retinal explants (**A**,**C**) and from explants after 3 days of incubation in the presence of *T. gondii* (**B**,**D**). The sections were counterstained with DAPI. Scale bar, 50 μm. See Figure 4 for abbreviations.

An additional consequence of inflammation in the retina is the increased expression of metalloproteinases (MMP). Indeed, we observed a relevant increase in MMP-2 immunofluorescence in retinal explants after 3 days of incubation, which was particularly intense within the IPL, OPL, and POS (Figure 8A,B). Inflammation is commonly observed together with oxidative stress, which we investigated using the marker 4-hydroxynonenal (4-HNE). While 4-HNE immunolabeling was very faint in control explants, it became evident throughout the retinal layers in 3-day infected explants, with particularly intense levels of immunofluorescence in the GCL and in the POS (Figure 8C,D).

### 3.3. Effects of T. gondii Infection on Retinal Cell Death

**Figure 9:**
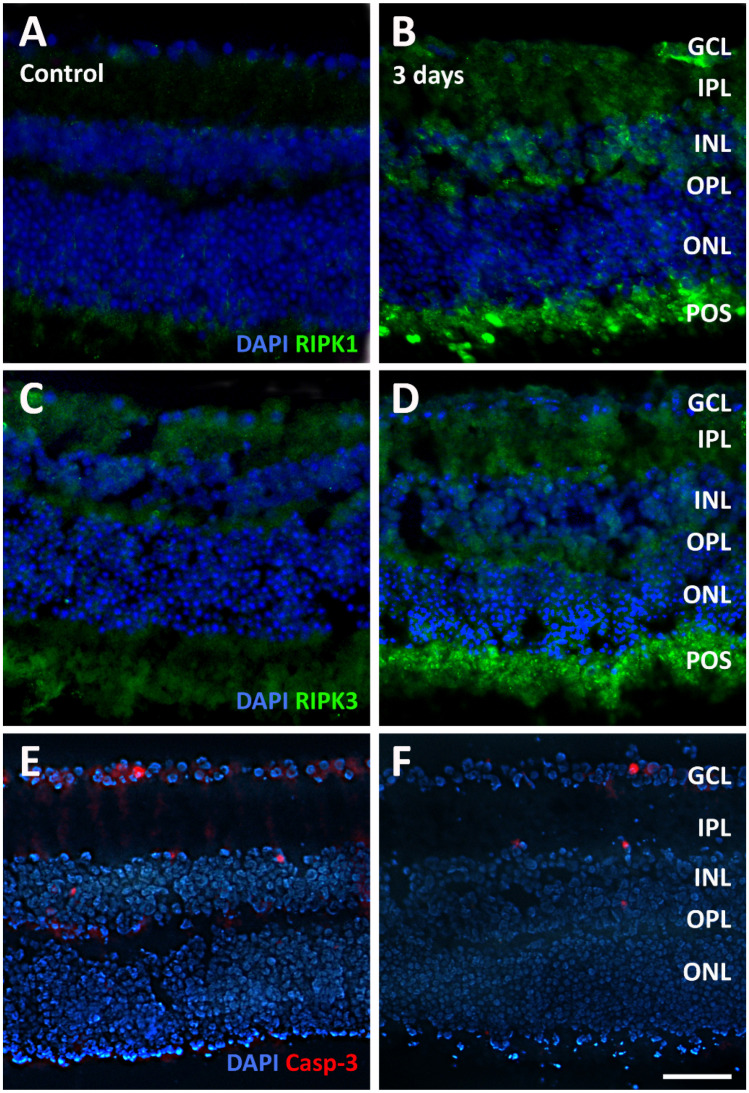
Representative photomicrographs of sections immunolabeled with an antibody directed to receptor-interacting serine/threonine kinase 1 (RIPK1, (**A**,**B**)), to RIPK3 (**C**,**D**) or to active caspase-3 (Casp-3) from control retinal explants (**A**,**C**,**E**) and from explants after 3 days of incubation in the presence of *T. gondii* (**B**,**D**,**F**). The sections were counterstained with DAPI. Scale bar, 50 μm. See Figure 4 for abbreviations.

Necroptosis is a type of cell death associated with inflammation. Receptor-interacting serine/threonine kinase 1 (RIPK1) and RIPK3 can be used as markers of necroptosis. RIPK1 immunolabeling was very faint in control sections (Figure 9A), while it was represented by brightly immunofluorescent puncta distributed in the inner retina (GCL, IPL, and INL), in the OPL, and in the POS of 3-day infected explants (Figure 9B). Similarly, low-level RIPK3 immunolabeling was observed in sections from control explants (Figure 9C), and a bright immunofluorescence signal was observed mainly in the POS of retinal explants after 3 days of incubation (Figure 9D). In contrast, no evident changes were observed in sections immunolabeled with antibodies directed to active caspase-3, a marker of apoptosis, where a few immunostained profiles could be seen in the GCL and in the INL of both control (Figure 9E) and 3-day infected (Figure 9F) explants.

## 4. Discussion

### 4.1. Comparison with Previous Models of OT

In vitro models of OT relied on human pigment epithelial or endothelial cell cultures, while other models have been established in vivo [4,6]. In vitro cell cultures are easy to manipulate; however, the retinal structure and the natural organic environment are lacking. On the other hand, in vivo experimental models may be challenging in different aspects (mainly logistics and ethics).

In an in vivo model of acute OT, the B1 gene of *T. gondii* was detected in the retina as early as 5 days post-injection, when onset of choroiditis could also be detected [17]. The evolution of choroiditis could be followed up to 20 days post-injection, while the actual presence of *T. gondii* within the retina was documented after 18 days post-injection. In our model, the parasites started retinal invasion very soon after incubation with the explants, and virtually all consequences of *T. gondii* infection in the retinal tissue were established as early as after 3 days of incubation.

### 4.2. Characterization of the Ex Vivo Mouse Model

*T. gondii* tachyzoites reach the retina through the choroidal vessels, which supply the outer retina, or through the retinal artery, which supplies the inner retina (see [18] for review). Our observations show that the parasite approaches the retinal explants both from the GCL and from the photoreceptor side, and from there, it proceeds to invade the retinal layers. Therefore, it seems that, although the explants are missing the normal retinal vasculature, this model may replicate the normal process of retinal infection by *T. gondii*.

Once in the retina, *T. gondii* tachyzoites convert into bradyzoites and encyst to establish a chronic and asymptomatic infection [19]. The RH strain of *T. gondii*, used in the present study, has been reported to have lost its capacity to form tissue cysts (see [20] for references); however, cyst formation seems to happen in our ex vivo model, in which it has been observed starting from 3 days of incubation. Indeed, tachyzoite/bradyzoite interconversion of the RH strain has been observed both in vitro and in vivo in different experimental conditions [20]. In addition, the putative cysts observed in our explants were immunolabeled by *T. gondii*-specific antibody, which, in previous studies, has been reported to stain *T. gondii* cysts in vitro [21]. Different from observations in vivo, where the cysts were preferentially localized to the GCL and IPL [22], in our model, the putative cysts did not show any layer preference, being observed throughout the retinal thickness.

The microglia in infected retinal explants seem to maintain a dynamic response. Indeed, the transitional state observed at 24 h and subsequent activation at 3 days suggests a time-dependent modulation of microglial activity in response to *T. gondii*. However, the nature of the interaction between *T. gondii* and microglial cells remains to be elucidated: on the one hand, microglial cells may become infected by *T. gondii*, while, on the other, they could phagocytose *T. gondii*. In favor of *T. gondii* phagocytosis by microglial cells, previous studies demonstrated that phagocytosis in microglial cells may be stimulated by proinflammatory cytokines via NO produced by iNOS [23,24]. In particular, iNOS upregulation concomitant with microglia activation has been observed in retinal inflammation occurring in glaucoma and diabetes [25,26,27]. Our findings indicating microglia activation and increased iNOS in infected explants align with these observations. The finding of microglia cells invaded by the parasites agrees with the results of in vivo studies carried out in orally infected BALB/c mice, where microglia cells, but not astrocytes, resulted in close interaction with the parasite, suggesting a role of microglia cells in chronic (two months of infection) ocular toxoplasmosis [22]. Similar to iNOS, eNOS is also likely to be implicated in retinal disease [28], being activated downstream of vascular endothelial growth factor [29] and affecting vascular permeability [30]. Our findings of increased eNOS immunoreactivity in retinal vessels and in the GCL of infected explants are similar to those observed in rat retinas suffering from oxidative stress induced by ischemia-reperfusion [31].

A typical feature of OT is the establishment of an inflammatory response. Indeed, in this study, it was possible to observe fully activated microglia after 3 days of infection. Our results also indicate a significant activation of NF-kB, an oxidant-sensitive transcription factor regulating the expression of other factors involved in inflammation [32], and an increase in the levels of IL-6, a key cytokine in both acute and chronic inflammation [33], demonstrating the establishment of an inflammatory response in infected explants. After *T. gondii* infection, mice lacking IL-6 were more prone to inflammation in their retina and vitreous body, with a higher parasite burden in their eyes compared to wild-type mice, suggesting a potential protective function of IL-6 [34]. Additionally, in vitro studies indicated that IL-6 might impede parasite multiplication by fostering encystment [35]. In our model, a significant elevation in IL-6 is noticeable after 3 days of incubation, which aligns with the proposed protective function of IL-6, potentially contributing to the subsequent putative encystment observed at 3 and 7 days of incubation. Inflammation in the retina is also accompanied by increased MMP-2 production, another marker of ocular inflammation [36]. MMP-2 expression is regulated by NF-kB [37], and macrophages infected by *T. gondii* express and release MMP to facilitate their migration [38,39]. Our observations reporting marked increases of MMP2 immunostaining in infected explants show that our model allows extensive investigation of the inflammatory response caused by *T. gondii*.

Inflammation and oxidative stress are likely to co-occur and interact in retinal pathologies [40]. 4-HNE is a product of lipid peroxidation. In the extracellular space, it may interact with membrane protein thiols [41] and is considered a reliable marker of oxidative stress [42,43]. The observation that 4-HNE immunofluorescence mostly stains the GCL and POS indicates that such a process occurs mainly in the retinal layers, which are the first to come in close contact with the parasites.

Cell death may occur through different mechanisms [44]. In particular, necroptosis is a form of regulated necrosis that is implicated in the pathogenesis of a variety of inflammatory diseases. Therefore, we investigated two markers of necroptosis such as RIPK1 and RIPK3 [45]. In addition, we also examined the apoptotic patterns using active caspase-3 as a marker. The data show that acute OT is likely to induce retinal cell death through necroptosis and not through apoptosis. This is particularly interesting since, as discussed above, OT is characterized by strong activation of inflammation, and necroptosis is known to be a highly proinflammatory mechanism, while apoptosis is thought to occur primarily without triggering inflammation [46,47]. Different from apoptosis, in necroptosis, cell membrane integrity is lost, triggering the activation of the immune system and inflammation [48].

### 4.3. Advantages and Shortcomings of the Proposed Model

Our model represents the application to OT of a well-known model previously used to study retinal changes in different pathological conditions [9,10,11,12,13,14]. The data provide enough evidence to establish this model as a suitable approach for the study of OT. Indeed, it has several advantages in comparison with previous models: (i) limited number of animals to be sacrificed; (ii) possibility to create different experimental conditions, such as infection with different amounts of parasites or different incubation times; (iii) possibility to study the modulation of the local immune response by different biological agents as well as the effects of anti-toxoplasmic drugs; (iv) the observation of putative cyst formation suggests that there are adequate conditions for tachyzoite/bradyzoite interconversion, and this aspect may also be studied in this model. On the other hand, it should be considered that the physiological conditions of the retina in vivo are only partially replicated in the ex vivo model; for instance, the explant does not receive blood supply and, therefore, possible effects of circulating elements, including endocrine factors, cannot be evaluated. In addition, although all of the main features of retinal infection by *T. gondii* were established within 3 days, the retinal explant model is unsuited for following the infection for periods of longer than 10 days due to unspecific damage that may occur after that period. Due to these limits, our results should be evaluated with some caution.

Some aspects of the proposed ex vivo experimental model may need some adjustment. For instance, the use of 2000 parasites/well added to the culture is likely to only partially reflect a natural infection. In an in vivo model of OT, 1 × 10^4^ tachyzoites were injected intraperitoneally [17], implying that a very small amount of them could arrive at the retina. Therefore, a refinement of our ex vivo model may require a dose-dependency study in which, starting with very small numbers, different amounts of parasites are added to the culture. Furthermore, future studies will need a quantitative analysis based on molecular tools.

### 4.4. Conclusions and Future Perspectives

The main advantages of the proposed model are the possibility to study the very early moments of parasitic invasion, to identify the retinal cell types that are mostly affected by the parasite, and to exploit possible effects of new drugs using a very limited number of animals. Together with other available models of OT, this new ex vivo model may contribute to further advances in the molecular and cellular mechanisms underlying this pathology and to new indications for potential therapeutic targets.

## Figures and Tables

**Table 1:** Antibodies used for immunofluorescence.

Antigen	Host Species	Mono/Polyclonal	Dilution	Source	Cat. No
*T. gondii* *	Goat	Polyclonal	1:100	Thermo Fisher Scientific, Waltham, MA, USA	PA1-7256
Iba1 *†	Rabbit	Monoclonal	1:1000	Abcam, Cambridge, UK	ab178846
iNOS †	Rabbit	Monoclonal	1:200	Abcam	ab178945
eNOS †	Rabbit	Polyclonal	1:200	Santa Cruz Biotechnology, Dallas, TX, USA	sc-654
MMP-2 †	Rabbit	Polyclonal	1:200	Santa Cruz Biotechnology	sc-10736
4-HNE †	Rabbit	Polyclonal	1:100	Kindly provided by Alfonso Pompella, University of Pisa	Not applicable
RIPK1 †	Rabbit	Polyclonal	1:100	Bio-Techne, Minneapolis, MA, USA	77077
RIPK3 †	Rabbit	Polyclonal	1:200	Bio-Techne	77299
Active caspase-3	Rabbit	Polyclonal	1:500	Cell Signaling Technology, Danvers, MA, USA	9664

* These antibodies were also used in double-label immunofluorescence experiments. † Abbreviations: 4-HNE, 4-Hydroxy-2-nonenal; Iba1, ionized calcium-binding adapter molecule 1; eNOS, endothelial nitric oxide synthase; iNOS, inducible nitric oxide synthase; MMP-2, matrix metalloproteinase-2; RIPK1, receptor interacting serine/threonine kinase 1; RIPK3, receptor interacting serine/threonine kinase 3.

**Table 2:** Antibodies used for Western blotting.

Antigen	Host Species	Mono/Polyclonal	Dilution	Source	Cat. No
β-actin	Mouse	Monoclonal	1:2500	Merck Sigma-Aldrich, Darmstadt, Germany	A2228
NF-kB p65 †	Rabbit	Polyclonal	1:1000	Abcam, Cambridge, UK	ab16502
pNF-kB p65 †	Rabbit	Polyclonal	1:100	Santa Cruz Biotechnology, Dallas, TX, USA	sc-33020
IL-6 †	Mouse	Monoclonal	1:3000	Santa Cruz	sc-57315

† Abbreviations: NF-kB p65, p65 subunit of nuclear factor kappa-light-chain-enhancer of activated B cells; pNF-kB p65, NF-kB p65 phosphorylated at Ser 276; IL-6, interleukin-6.

## Data Availability

Data are contained within the article.

## Supplementary Materials

The following supporting information can be downloaded at: https://www.mdpi.com/article/10.3390/pathogens13080701/s1, Figure S1: Representative photomicrographs of Vero cells immunolabeled with an antibody directed to *T. gondii*. (A–C): Immunolabeled cells from a control, non-infected culture; (D–E): immunolabeled cells from an infected culture. (A,D) DAPI nuclear staining; (B,E), *T. gondii* immunofluorescence; (C,F) overlay of the corresponding DAPI and *T. gondii* staining. Scale bar, 70 μm.
