# Correction: Rodriguez Fernandez et al. A New Ex Vivo Model Based on Mouse Retinal Explants for the Study of Ocular Toxoplasmosis. *Pathogens* 2024, *13*, 701

**DOI:** 10.3390/pathogens14121229

**Published:** 2025-12-02

**Authors:** Veronica Rodriguez Fernandez, Rosario Amato, Simona Piaggi, Barbara Pinto, Giovanni Casini, Fabrizio Bruschi

**Affiliations:** 1 Department of Translational Research, School of Medicine, University of Pisa, 56126 Pisa, Italy; veronica.rodriguezfernandez@uniroma1.it (V.R.F.); barbara.pinto@unipi.it (B.P.); 2 Department of Infectious Diseases and Public Health, La Sapienza University, 00185 Rome, Italy; 3 Department of Biology, University of Pisa, 56126 Pisa, Italy; 4 Interdepartmental Research Center Nutrafood “Nutraceuticals and Food for Health”, University of Pisa, 56126 Pisa, Italy

In the original publication [1], there was a mistake in Figure 9 as published. The mistake was that a preliminary version of the figure was used inadvertently in place of the correct Figure 9. The preliminary figure included overlaps in the DAPI backgrounds of panels B, E, and F. The correct Figure 9 appears below:

The authors state that the scientific conclusions are unaffected. This correction was approved by the Academic Editor. The original publication has also been updated.

## Figures and Tables

**Figure 9:**
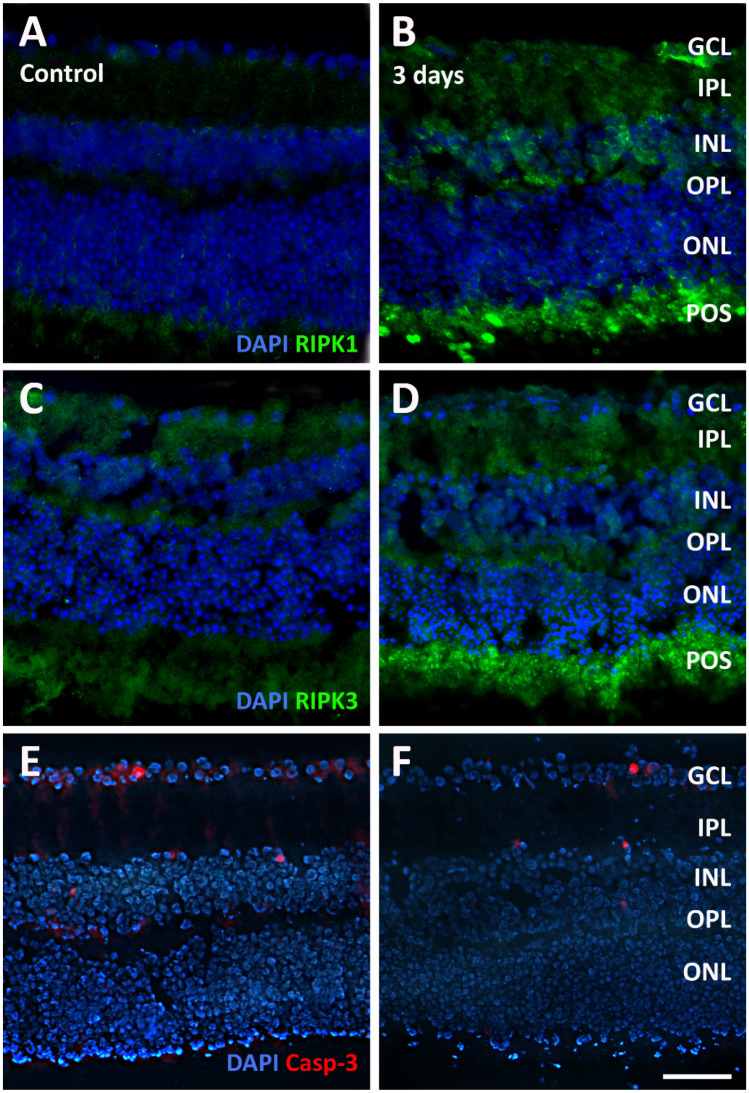
Representative photomicrographs of sections immunolabeled with an antibody directed to receptor-interacting serine/threonine kinase 1 (RIPK1, (**A**,**B**)), to RIPK3 (**C**,**D**) or to active caspase-3 (Casp-3) from control retinal explants (**A**,**C**,**E**) and from explants after 3 days of incubation in the presence of *T. gondii* (**B**,**D**,**F**). The sections were counterstained with DAPI. Scale bar, 50 μm. See Figure 4 for abbreviations.
