# Supplementary material for: A New Ex Vivo Model Based on Mouse Retinal Explants for the Study of Ocular Toxoplasmosis
Source: Pathogens. 2024 Aug 19;13(8):701. doi: 10.3390/pathogens13080701 (PMC11356793; doi:10.3390/pathogens13080701)
Supplement: Supplementary file 1 [file pathogens-13-00701-s001.zip › pathogens-3102832-supplementary.pdf]

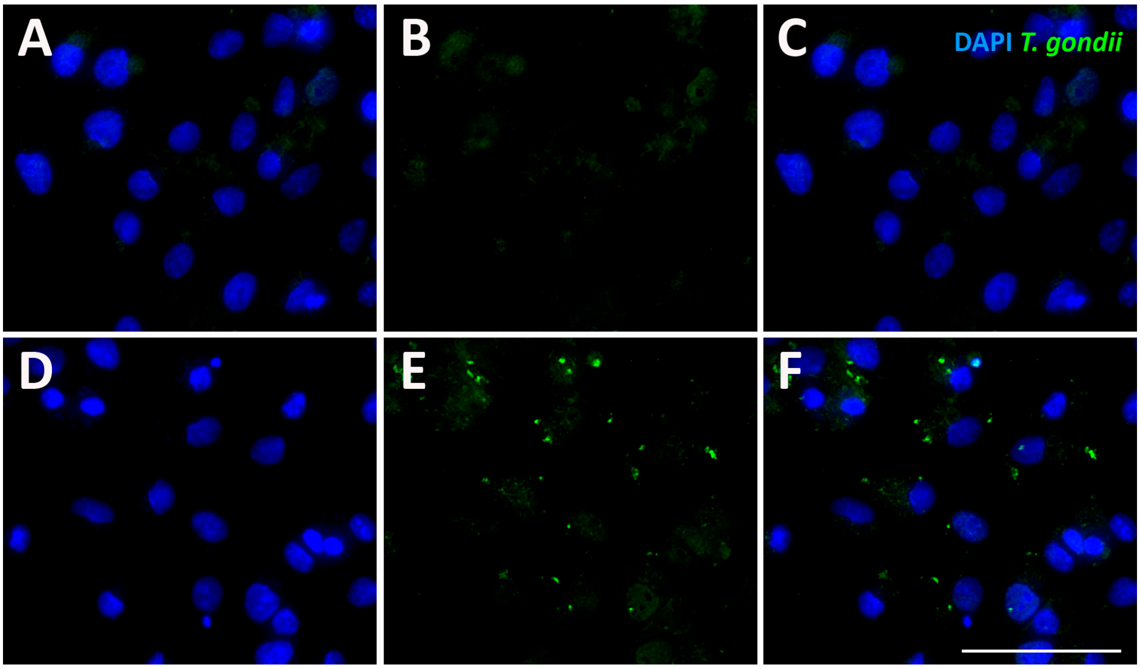

Figure S1. Representative photomicrographs of Vero cells immunolabeled with an antibody directed to *T. gondii*. (A–C): Immunolabeled cells from a control, non-infected culture; (D–E): immunolabeled cells from an infected culture. (A,D) DAPI nuclear staining; (B,E), *T. gondii* immunofluorescence; (C,F) overlay of the corresponding DAPI and *T. gondii* staining. Scale bar, 70 μm.
